# Communication style and exercise compliance in physiotherapy (CONNECT). A cluster randomized controlled trial to test a theory-based intervention to increase chronic low back pain patients’ adherence to physiotherapists’ recommendations: study rationale, design, and methods

**DOI:** 10.1186/1471-2474-13-104

**Published:** 2012-06-15

**Authors:** Chris Lonsdale, Amanda M Hall, Geoffrey C Williams, Suzanne M McDonough, Nikos Ntoumanis, Aileen Murray, Deirdre A Hurley

**Affiliations:** 1 School of Science and Health, University of Western Sydney, Locked Bag 1797, Penrith, NSW, 2751, Australia; 2 School of Public Health, Physiotherapy, and Population Science, Health Sciences Centre, University College Dublin, Belfield, Dublin 4, Ireland; 3 Departments of Medicine, Clinical and Social Sciences in Psychology, Psychiatry, Center of Community Health, University of Rochester, 46 Prince St Suite 3001, Rochester, NY, 14607, USA; 4 Health and Rehabilitation Sciences Research Centre, School of Health Sciences, University of Ulster, Ulster, BT37 0QB, UK; 5 School of Sport and Exercise Sciences, University of Birmingham, Edgbaston, Birmingham, B15 2TT, UK

**Keywords:** Communication, Motivation, Patient, Low back pain, Disability, Well-being, Adherence, Compliance, Physical therapy, Physiotherapy

## Abstract

**Background:**

Physical activity and exercise therapy are among the accepted clinical rehabilitation guidelines and are recommended self-management strategies for chronic low back pain. However, many back pain sufferers do not adhere to their physiotherapist’s recommendations. Poor patient adherence may decrease the effectiveness of advice and home-based rehabilitation exercises. According to self-determination theory, support from health care practitioners can promote patients’ autonomous motivation and greater long-term behavioral persistence (e.g., adherence to physiotherapists’ recommendations). The aim of this trial is to assess the effect of an intervention designed to increase physiotherapists’ autonomy-supportive communication on low back pain patients’ adherence to physical activity and exercise therapy recommendations.

**Methods/Design:**

This study will be a single-blinded cluster randomized controlled trial. Outpatient physiotherapy centers (*N* =12) in Dublin, Ireland (population = 1.25 million) will be randomly assigned using a computer-generated algorithm to either the experimental or control arm. Physiotherapists in the experimental arm (two hospitals and four primary care clinics) will attend eight hours of communication skills training. Training will include handouts, workbooks, video examples, role-play, and discussion designed to teach physiotherapists how to communicate in a manner that promotes autonomous patient motivation. Physiotherapists in the waitlist control arm (two hospitals and four primary care clinics) will not receive this training. Participants (*N* = 292) with chronic low back pain will complete assessments at baseline, as well as 1 week, 4 weeks, 12 weeks, and 24 weeks after their first physiotherapy appointment. Primary outcomes will include adherence to physiotherapy recommendations, as well as low back pain, function, and well-being. Participants will be blinded to treatment allocation, as they will not be told if their physiotherapist has received the communication skills training. Outcome assessors will also be blinded.

We will use linear mixed modeling to test between arm differences both in the mean levels and the rates of change of the outcome variables. We will employ structural equation modeling to examine the process of change, including hypothesized mediation effects.

**Discussion:**

This trial will be the first to test the effect of a self-determination theory-based communication skills training program for physiotherapists on their low back pain patients’ adherence to rehabilitation recommendations.

**Trial Registration:**

Current Controlled Trials ISRCTN63723433

## Background

Low back pain is a significant global problem, with up to 85% of the population in developed countries experiencing an acute episode at some point in their lifetime [1]. A significant number of these patients develop chronic low back pain (CLBP), defined by persistent disabling pain in the lumbar spine, with or without radiation to the buttock and lower limbs [2], for more than 12 weeks [3]. In addition to pain complaints, CLBP is associated with reduced physical function, reduced social participation, increased symptoms of psychological distress, and poorer quality of life [4]. It is also an increasingly costly condition, due to the expense of treatment and lost productivity. Accounting for 0.8 to 2.1% of gross domestic product in many western countries [5], CLBP is estimated to be the second largest single cause of work absence in the United Kingdom [6]. As a result, the efficacy of treatments designed to alleviate CLBP has been the subject of much scientific attention [7].

Physical activity (PA) and exercise therapy (i.e., specific repetitive movements intended to reduce LBP) [7] are among the accepted clinical rehabilitation guidelines and are recommended self-management strategies [8] for this condition. However, many LBP sufferers do not adhere to their physiotherapist’s recommendations regarding PA and exercises [9,10]. Poor patient adherence may decrease the effectiveness of PA advice and home-based rehabilitation exercises [11]. Therefore, interventions that can increase patients’ adherence may also enhance treatment outcomes [12].

Theory-based interventions are needed in the health domain [13], as they provide greater understanding of the process of change and may ultimately lead to more effective interventions [14,15]. A recent Cochrane systematic review indicated that there was support for therapeutic interventions designed to increase adherence to treatments for musculoskeletal pain conditions [16]. Indeed, the review found moderate sized effects on patients’ adherence. Unfortunately, only two of these interventions [17,18] were based on a relevant behavior change theory that might explain the process of change resulting from the intervention. Therefore, the most effective methods to increase adherence and the active components of the majority of these interventions remain unclear. Recommendations from recent research [19,20] and the Medical Research Council [15] have reiterated the importance of (i) using theory and, where possible, empirical evidence to guide the development of interventions and (ii) investigating treatment fidelity and the process of change to allow researchers to provide effective advice for successful implementation into practice.

A theory-based intervention to improve adherence should aim to address factors that influence chronic low back pain patients’ rehabilitation behavior. Research indicates that these factors may include (i) the physiotherapist-patient relationship [21,22], (ii) the delivery of advice [23], self-efficacy [19,24], and motivation for treatment [25,26]. Self-determination theory (SDT) [27] may provide a useful framework for addressing these factors, thereby increasing treatment adherence, and improving patient outcomes.

### Self-determination theory

**Figure 1:**
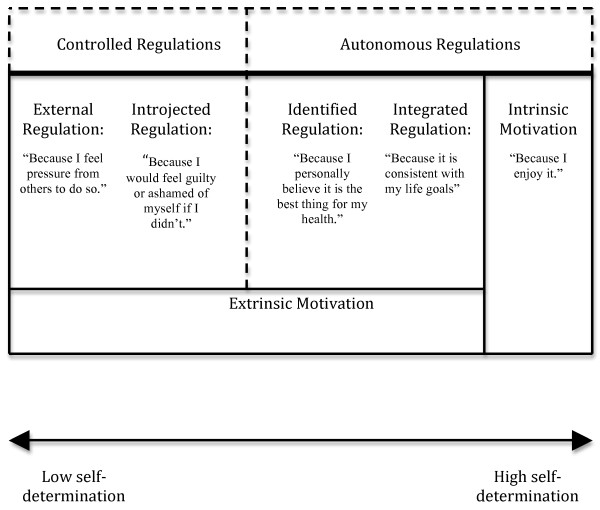
The Self-Determination Continuum of Motivation (with examples quotes to illustrate motives for following a physiotherapist’s recommendations).

According to SDT [27], humans have basic psychological needs for autonomy (feeling fully volitional or free to engage in a behavior), perceived competence (feeling effective in one’s actions), and relatedness (feeling safe and cared for in one’s interpersonal relationships). When these needs are supported, patients’ participation in treatment will be more autonomous and less controlled. Autonomous motivation is characterized by perceptions of valued benefits and a willingness to participate. In contrast, controlled motivation in the healthcare domain typically involves patient engagement in treatment due to external pressure, coercion, or feelings of guilt. This distinction between autonomous and controlled motivation represents a continuum rather than a dichotomy (see Figure 1 for details), with more autonomously motivated behaviors leading to greater psychological well-being and long-term behavioral persistence [28]. 

When the social context (i.e., interactions with other people) satisfies the three basic psychological needs, individuals are more likely to autonomously regulate their behaviors, and thus lasting behavior change is more likely [28]. In health-related domains, this suggests that healthcare practitioners’ communication behaviors can be enhanced to more fully support patients’ psychological needs and, thereby, autonomously motivate their health–related behaviors. In this context, the concept of autonomy support represents an interpersonal climate in which the provider (e.g., physiotherapist) considers the perspective of the patient, provides relevant information and opportunities for choice, and encourages the individual to accept personal responsibility for health behaviors without judging or coercing the patient (see further examples in method section) [27]. In contrast, a controlling health care climate involves disregarding patients’ views, pressuring patients, and making the decisions on the patients’ behalf without consultation. Unfortunately, research has indicated that when interacting with patients, physiotherapists [21] and other healthcare practitioners [29] often adopt a controlling approach.

**Figure 2:**
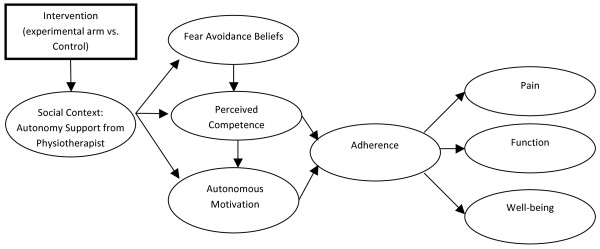
Self-Determination Theory Model of Behavior Change.

In line with the SDT-based model of health behavior change [30,31], the relationship between the healthcare provider’s autonomy support and the patient’s behavior change via autonomous motivation and perceived competence has been supported in numerous health settings including smoking cessation [30], physical activity [32], medication adherence [33], and dental hygiene [34]. Evidence from cohort studies in physiotherapy settings has supported the positive relationship between autonomy support and adherence outcomes, such as attendance at clinic-based rehabilitation settings [35] and adherence to home-based exercise programs [26], However, no study has been conducted to test the effect of an intervention designed to enable physiotherapists to act in a more autonomy supportive manner during the therapeutic scenario. This type of intervention could increase CLBP patients’ autonomous motivation and competence leading to improved adherence to prescribed home-based treatment and improved LBP outcomes. A diagram presenting the proposed theoretical model of behavior change is presented in Figure 2. 

We conducted a pilot study [36] to establish if an SDT-based intervention designed to enhance physiotherapists’ communication skills had the capacity to influence the targeted variable (autonomy support) and produce change in the proposed mediators (perceived competence and autonomous motivation), as well as treatment adherence for CLBP patients attending physiotherapy. The findings suggested that the SDT-based training enhanced physiotherapists’ autonomy supportive communication skills and provided initial evidence that the intervention improved patients’ perceived competence, autonomous motivation, and treatment adherence. However, the study was limited by the small sample and a number of study design factors, such as unexpected between group differences in the duration of patients’ LBP and the lack of true baseline measures. Nonetheless, these preliminary results were positive and provided estimates of moderate sized effects. The proposed study is an extension of the pilot study and addresses the above limitations.

### Aims

The aim of this cluster randomized controlled trial (RCT) is to assess the effect of an intervention designed to increase physiotherapists’ autonomy-supportive communication on CLBP patient’s adherence to physical activity and LBP exercise recommendations.

### Hypotheses

1. Patients in the experimental arm will report significantly greater weekly physical activity (PA) participation compared with their pre-treatment PA levels and compared with patients in the control arm. They will also report greater self-rated adherence to physiotherapists’ recommendations compared with the patients in the control arm. Compared with physiotherapists in the control arm, physiotherapists in the experimental arm will rate their patients as more adherent during physiotherapy sessions.

2. Patients in the experimental arm will report significantly decreased pain, increased function, greater low back pain (LBP)-related well-being and greater perceived global improvement after treatment compared with their pre-intervention scores, and compared to patients in the control arm.

3. Compared with their pre-treatment scores and compared with patients in the control arm, patients in the experimental arm will report significantly lower fear-avoidance beliefs and controlled motivation, as well as significantly greater competence and autonomous motivation.

4. Patients in the experimental arm will rate their physiotherapists as significantly more autonomy supportive than patients whose physiotherapists were assigned to the control arm. As a result of heightened self-awareness that comes from participation in communication skills training, physiotherapists assigned to the experimental arm will rate themselves as less autonomy supportive than physiotherapists assigned to the control arm. Independent raters of audio recordings of patient-physiotherapist interactions will rate experimental arm physiotherapists as more autonomy supportive than physiotherapists assigned to the control arm. Physiotherapists in the experimental arm will employ the specific communication strategies taught in the workshops with higher quality than physiotherapists from the control arm (who will not attend these workshops).

5. The influence of the experimental manipulation on outcomes (pain, function, and well being) will be mediated by patients’ rating of the physiotherapist’s autonomy support, perceived competence, autonomous motivation, fear-avoidance beliefs and adherence (see Figure 2).

## Methods/Design

### Design overview

**Figure 3:**
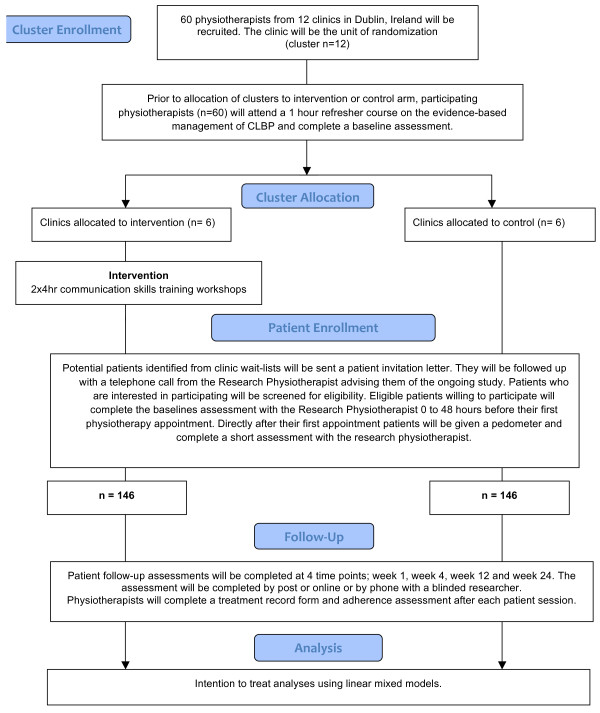
CONSORT 2010 flow diagram.

This study will be a single-blinded cluster RCT. Physiotherapists in the experimental arm (two hospitals and four primary care clinics) will attend eight hours of communication skills training. Physiotherapists in the waitlist control arm (two hospitals and four primary care clinics) will not receive this training. Participant assessments will be conducted by researchers blinded to treatment allocation and will occur at baseline, as well as 1 week, 4 weeks, 12 weeks, and 24 weeks after their first physiotherapy appointment (see Figure 3 for an overview). The primary endpoint for analysis will be data collected at week 24. Recruitment is expected to take place from April 1, 2011 to June 15, 2012.

### Participant recruitment, consent, allocation and blinding

#### Centers

Managers of 12 publicly funded outpatient physiotherapy centers in the Dublin, Ireland area (population = 1.25 million) have agreed to participate. Centers include outpatient physiotherapy departments located in the four largest hospitals in the city and all eight primary community and continuing care clinics (hereafter referred to as “community clinics”) in the greater Dublin area. Centers will be assigned to the experimental or control arm after they have agreed to participate in the study, so the researchers (DAH and AH) who recruited the centers will be blinded to allocation. To assign the four hospitals to the two conditions (1:1), a person blinded to the purposes of the study will use a computerized random number generator algorithm. The same procedure will be used to randomly assign (1:1) the eight community clinics to the two conditions. A researcher (CL) will contact all physiotherapists to inform them of their allocation arm. Experimental arm physiotherapists will begin the communication skills training workshops within two weeks of allocation. Control arm physiotherapists will be offered the opportunity to complete the training at the end of the trial (i.e., waitlist control).

Investigators responsible for the monitoring of treatment and attending to clinical issues that arise during the conduct of the study will be unblinded. Investigators responsible for data collection will be blinded to treatment allocation for the duration of trial. The investigators responsible for data analysis will use a coded dataset to ensure blinding.

Ethical approval has been granted by Research Ethics Committees that cover all recruitment sites. These include the (i) Adelaide and Meath Hospital Ethics Committee, (ii) Connolly Hospital Ethics Committee, (iii) Beaumont Hospital Ethics Committee, (iv) St. Vincent’s University Hospital Ethics Committee, and (v) Health Service Executive for Dublin Primary Care Community Clinics (Kildare, West Wicklow, Dublin SouthWest, North Dublin, Dublin North City, Dun Laoghaire, Dublin South East, Dublin South West, Wicklow, and Dublin South City).

#### Physiotherapists

All physiotherapists at participating sites will be informed that the trial is designed to evaluate the effect of a communication skills training program and will be invited to participate in the study. All physiotherapists practicing in publicly funded clinical settings in Ireland must be Chartered Physiotherapists. No further inclusion or exclusion criteria will be specified. Those who agree to participate will be asked to provide written consent, complete a baseline assessment, and attend a one hour education session (“Evidence-based care for CLBP management refresher workshop”). Physiotherapists will not be blinded to treatment allocation, as they are implementing the intervention with patients.

#### Patients

Patients with CLBP will constitute the patient sample. As randomization will be by center, all participants in a given center who agree to participate will either belong to the experimental arm or the control arm. Each patient referred by a medical practitioner for physiotherapy for CLBP to a public hospital or community clinic will receive a detailed letter and patient information leaflet outlining the purpose of the study. The letter will be followed by a phone call from the Research Physiotherapist inviting the patient to participate and confirming eligibility using the inclusion/exclusion criteria listed in Table 1. Patients who meet all the inclusion criteria will receive detailed verbal information about the trial and then will be asked to provide verbal consent to participate in the study. Written informed consent will be collected prior to baseline assessment. To ensure unbiased behavior and assessments, participants will be blinded to treatment allocation, as they will not be told if their physiotherapist has received the communication skills training.

**Table 1:** Inclusion and exclusion criteria


*Inclusion criteria*	
Age	18 to 70 years
Diagnosis	LBP of mechanical origin with/ without radiation to the lower limb
Pain duration	chronic (≥3 months) or recurrent (≥3 episodes in previous year)
Language	English speaking and English literate.
Contact status	Access to a telephone
*Exclusion criteria*	
Pathology	Suspected or confirmed serious spinal pathology (fracture, metastatic, inflammatory or infective diseases of the spine, cauda equina syndrome/widespread neurological disorder).
	Nerve root compromise (2 of strength, reflex or sensation affected for same nerve root)
Past medical history	Spinal surgery or History of systemic / inflammatory disease
Current medical status	Scheduled for major surgery during treatment
Treatment status	Currently or having received treatment for CLBP within previous 3 months
Pregnancy	Suspected or confirmed pregnancy
Contraindications	Unstable angina / uncontrolled cardiac dysrhythmias / severe aortic stenosis / acute systemic infection accompanied by fever. No confounding conditions, such as a neurological disorder, intellectual disorder.
Note: Individuals suspected of having a serious spinal pathology or any contraindication to exercise will be referred to their medical practitioner for review. Once cleared by their medical practitioner they will be reconsidered for inclusion in the trial.	

### Interventions

#### Evidence-based care for CLBP management refresher workshop

In both the experimental and control arms, physiotherapists will participate in a one-hour refresher workshop on evidence-based physiotherapy care for CLBP, including recommendations to include PA as part of home-based rehabilitation. This workshop will be delivered by a research investigator (DAH) who holds a PhD in back pain research in Physiotherapy. The physiotherapists will also be provided with a one-page summary of the recommended advice to patients based on current evidence-based clinical guidelines for CLBP. The purpose of this session is not to teach physiotherapists new interventions, but to provide a brief reminder of current standard practice guidelines and help ensure that recommendations provided by physiotherapists are similar between arms. This session will also offer the opportunity to answer any questions physiotherapists may have regarding the study protocol and data-collection. Additionally, the Research Physiotherapist will use the end of the workshop to provide instruction on how to complete the treatment record and adherence assessment used in data collection for every included patient (see assessment procedures).

#### *Experimental Treatment – Theory-based Communication Skills Training Workshops* (CONNECT)

In addition to the evidence-based CLBP care refresher workshop, the physiotherapists in the experimental arm will complete eight hours of communication skills training. A research investigator (CL) who holds a PhD in Sport and Exercise Psychology will deliver these training workshops based on self-determination theory principles, implemented via the ‘5A’ framework (ask, advise, agree, assist, arrange) [32,37].

Within each of the ‘A’s of the 5A framework (as implemented in CONNECT), there are a number of strategies that have been specifically adapted to suit a physiotherapy session for patients with CLBP. A description of each strategy and the manner in which it maps onto constructs from SDT are provided in Table 2. Briefly, the ‘Ask’ phase largely involves strategies designed to promote patients’ sense of relatedness. During the ‘Advise’ phase physiotherapists are taught to build patients’ sense of autonomy, while ensuring they understand the nature of their LBP and treatment options that are most likely to be effective (enhancing perceived competence). In the ‘Agree’ stage, physiotherapists learn to collaboratively set ‘SMART’ goals that will enhance patient autonomy and help them believe that these goals are achievable (enhancing competence). In the ‘Assist’ phase, competence and autonomy are fostered by helping the patient to identify likely obstacles to adherence, as well as potential methods to overcome these challenges. Finally, in the ‘Arrange’ stage, physiotherapists are taught to provide a rehabilitation diary to support patients’ competence and autonomy, and ensure that patients feel the physiotherapists wants to ensure that they have adequate resources to support their home-based rehabilitation (enhancing relatedness and competence).

**Table 2:** Mapping communication strategies to the ‘5A’ framework and self-determination theory

**Strategy**	**Description / Example**	**Main Basic Psychological Need(s) Targeted**
**ASK**		
Using Open-Ended Questions	“Tell me”/“What”/”How” are useful terms when asking questions, as they allow the patient to elaborate on his/her story. Example: *“What kind of things are you doing to alleviate the pain at the moment”*	Relatedness
Using Single Questions	Avoid asking multiple questions at one time. Instead, ask one question and wait for a response before asking a second question.	Relatedness
Staying Silent	Allow the patient to complete sentences and finish speaking before following up with further questions.	Relatedness
Paraphrasing	After listening to the patient, summarize your perception of the main points. Examples: “*So what I am hearing is that…” or “It sounds like …”*	Relatedness
Empathizing	Show the patient that you understood the emotions that went along with the issue being discussed. Examples: *“I can see this upsets you” or “That must be very frustrating”.*	Relatedness
Gauging Patient Readiness to accept advice	Ask the patient if he or she is ready to consider advice regarding activities outside the clinic. Example: *“There a number of things you can do that will help … would you like to hear a few suggestions?”*	Autonomy
**ADVISE**		
Catering for Different Learning Preferences	Use a selection of methods (aural, visual, kinesthetic) to educate the patient (during session and take home materials); these methods cater for multiple learning preferences.	Competence
Closing the Loop	Ask patients to paraphrase/demonstrate information that had been provided. Provide corrective feedback as required, and re-test understanding. Example: “*To be sure that I was clear, could you please tell me, in your own words, your understanding of the …”*	Competence
Providing a Rationale	Explain to the patient the rationale behind your advice. Example: *“As we discussed earlier, your back needs support from the muscles around. So, if you can do these exercises, you can really provide your back with extra support …” or “Research shows that PA, such as walking, is a great way to…”*	Autonomy
Providing Opportunities for Patient Input or Choice	Ask the patient to provide input or make choices when providing advice. Example: “*Getting some physical activity –like going for a walk, riding your bike or swimming – is really good for your back. Is there a type of exercise that you prefer?”*	Autonomy
Using Autonomy Supportive Phrases Instead of Controlling Language	Support and encourage the patient to accept personal responsibility for his/her recovery. Avoid coercion or guilt inducing phrases. Examples: *“Here are some things that will help you overcome…” or “If you complete these exercises then you’ll strengthen your back and it will be less likely to give you pain”, instead of “Do this for me*” or “You have to…” or “You must…”.	Autonomy & Competence
**AGREE**		
Employing SMART Goal Setting	Agreed on goals that are Specific, Measurable, Achievable, Recorded, and Time-based. Example: *Earlier you mentioned that you are finding it hard walking for long periods. For this week we could set a target of 15 minutes walking per day, how many days do you think you couldachieve that target in the next week?”*	Competence
Ensuring Active Patient Participation in Goal Setting	Ask the patient for his/her opinions/comments during goal setting.Take into account patient’s subjective history (e.g. family/work commitments). Example: *What time of day would suit you best for these exercises?*	Autonomy & Competence
**ASSIST**		
Identifying Barriers and Obstacles	Discuss at least one likely barrier to following treatment advice. Example: “*Is there anything you can think of that might stop you from accomplishing your exercise goal?”*	Competence &Autonomy
Identifying Solutions and Obstacles	Brainstorm with the patient ways to overcome this barrier (e.g. ‘*identifying enablers’* and ‘cognitive restructuring’). Examples: “*Walking can be a fun and social activity that doesn’t seem like hard work. How would you feel about walking with a friend/neighbor?” and suggest changing thoughts from “I am too out of shape to walk to the shop” to “If I take it nice and easy and remember to breathe, relax and take a rest when I need one, I will be able to walk to the shop.”*	Competence & Autonomy
**ARRANGE**		
Providing a Rehabilitation Diary	Provide the patient with a rehabilitation diary to help him/her keep track of home-based rehabilitation (e.g., exercise, physical activity).	Competence & Autonomy
Following-Up	Suggest a specific follow-up appointment, provide guidance regarding when an appointment should be arranged (e.g., no more than 2 weeks later), or inform the patient that no follow-up appointment is needed.	Relatedness & Competence
Offering Contact	Invite the patient to contact you in the event of difficulties or questions.	Relatedness & Competence

Training will include handouts, workbooks, video examples, role-play, and discussion. These training methods (apart from the video examples) were successfully piloted in two Dublin-area physiotherapy clinics in 2009 [36]. At the end of each session, each physiotherapist will work with the workshop leader (CL) to set goals for strategy implementation during treatment sessions. The goals along with likely obstacles and solutions to overcome will be recorded. Physiotherapists in the experimental arm will receive individual follow-up emails 4 weeks and 10 weeks after the completion of training. During these follow-ups, the workshop leader will discuss progress towards the attainment of the implementation goals and provide assistance to resolve any problems physiotherapists may encounter when employing autonomy supportive communication in their clinical practice. Finally, treatment record forms for physiotherapists in the experimental arm will include 5A prompts, with specific reminders to use strategies taught during the communication skills workshops.

### Assessment

#### Physiotherapists

##### Baseline assessment

Prior to randomization, all physiotherapists participating in the study will be asked to complete a series of questionnaires (10 minutes long). These questionnaires are listed in Appendix A and will be completed prior to the “Evidence-based care for CLBP management refresher workshop” (see intervention section).

##### Treatment phase assessment

All physiotherapists will be provided with a Treatment Record and Adherence Assessment for each patient. This is a two-page assessment that will be completed by the physiotherapist (3–5 minutes) after each treatment session.

#### Patients

##### Baseline assessment

A schematic view of the assessment time line for patients is presented in Table 3. The baseline assessment is a self-report questionnaire regarding the patient’s demographic data, LBP history, primary and secondary outcome data, as well as assessments of potential moderating variables. The assessment will take approximately 20 minutes to complete and will be completed at the physiotherapy site administered by the Research Physiotherapist or in cases in which this does not suit the patient’s schedule, he/she can complete the assessment by telephone (maximum 48 hours before his/her physiotherapy appointment). Directly after the initial physiotherapy appointment, the patient will be given a pedometer and asked to complete two short questionnaires about his/her perceptions of the physiotherapist’s autonomy support and the patient’s motivation to follow the physiotherapist’s advice.

**Table 3:** Outcome assessment timeline

**Variable**	**Pre-randomization**	**Baseline Pre-treatment**	**Baseline Post-treatment**	**Week1**	**Week4**	**Week12**	**Week24**
**Demographics**		✓					
**Primary outcome measures**							
**Adherence**							
Clinic-based adherence to physiotherapist’s recommendations			#	#	#	#	
General adherence to physiotherapist‘s recommendations				✓	✓	✓	✓
Specific adherence to back exercises and physical activity advice				✓	✓	✓	✓
**Physical Activity**							
Self-reported physical activity		✓		✓	✓	✓	✓
**Low Back Pain Symptoms**							
Pain Intensity		✓			✓	✓	✓
Bothersomeness		✓			✓	✓	✓
**Pain-related Function**							
Disability		✓			✓	✓	✓
Patient specific function		✓			✓	✓	✓
**Pain-related Well Being**							
Quality of life		✓			✓	✓	✓
**Secondary Outcomes**							
Autonomy support from physiotherapist			*✓		✓		
Fear avoidance beliefs regarding physical activity		✓			✓	✓	✓
Perceived competence regarding ability to follow physiotherapist’s recommendations		✓	✓		✓	✓	✓
Autonomous and controlled motivation to following physiotherapist’s recommendations		✓	✓		✓	✓	✓
Objectively measured physical activity				✓	✓	✓	✓
Perception of recovery		✓			✓	✓	✓
**Moderating Variables**							
Expectation of treatment	*		✓				
Patient depression		✓					
Physiotherapist’s general causality orientations	*						
Physiotherapist’s autonomous and controlled motivation for participation in training.	*						

Note: ✓ = patient rated assessment. * = physiotherapist rated assessment. # = physiotherapist rating of patient behavior following each treatment session (most likely to occur during first 12 weeks following initial session).

##### Follow-up assessments (Week 1)

At week 1, after the patient’s initial physiotherapy session, he/she will be contacted by a blinded researcher to record the pedometer step count for the previous seven days, self-reported PA, and adherence. This will be done by a telephone call or an email, depending on the patient’s preference (provided at the time of recruitment). At this time, the patient can ask any questions regarding the use of their pedometer or any other general questions pertaining to their involvement in the study.

##### Follow-up assessments (Weeks 4, 12, and 24)

Follow-up outcome measures at Weeks 4, 12, and 24 will be collected via one of three methods, depending on patient preference; (i) over the telephone with a blinded research investigator, (ii) online questionnaire or (iii) hardcopy of the questionnaire sent via post with a pre-paid return envelope. Attempts to ensure complete follow-up data is collected on time will involve; (i) pre-posting the follow-up questionnaire to the patient 1 week before it is due with a pre-paid self-addressed return envelope for easy return postage, (ii) sending a text message to let the patient know the follow-up assessment has been posted, (iii) the Research Physiotherapist telephoning the patient during the week the assessment is due to ensure the patients has received the questionnaire and answer any questions he or she may have. In situations where the assessment is overdue by two weeks, the Research Physiotherapist will contact the patient by telephone and, if contact is not made, she will send a handwritten letter asking the patient to complete and return the questionnaire.

### Outcomes

A brief description of the outcomes is listed below. The timeline of assessments is detailed in Table 3. Detailed descriptions and references for these measures are presented in Appendix A.

#### Primary outcome measures

(i) *General adherence to physiotherapy recommendations*, during the therapy session as rated by the physiotherapist [adapted version of Sports Injury Rehabilitation Adherence Scale] and outside the clinic as rated by the patient [Adherence to Physiotherapists’ Recommendations Scale]. We will also collect information regarding the patient’s recall and adherence to recommendations regarding specific back exercises and PA using an adapted version of a Home Exercise Compliance Assessment.

(ii) *PA level,* self-reported using the International Short Form Physical Activity Questionnaire.

(iii) *Back Pain Symptoms, Function and Well Being,* measured by Pain Intensity [Numerical Rating Scale], Pain Bothersomeness, the Roland Morris Disability Questionnaire, the Patient Specific Functional Scale, the European Quality of Life Questionnaire, and the Perception of Recovery Scale.

#### Secondary outcome measures

We will employ secondary outcomes that we expect to change as a result of the intervention and could explain the effect of the intervention on the primary outcomes; including the measurement of autonomy support, perceived competence, autonomous and controlled motivation, as well as fear avoidance as it relates to physical activity.

#### Moderating variables

We will also measure specific individual factors at baseline that could influence the treatment effect. These include the therapist’s personality (causality orientations) and motivation to participate in the study, the therapist and patient’s treatment expectations, and the patient’s baseline levels of depressive symptoms. Descriptions and references for these measures are presented in Appendix A.

### Treatment fidelity

Audio-recordings will be used to assess fidelity of treatment. For this outcome, at least 20% of the physiotherapists in the control arm and at least 20% of physiotherapists in the experimental arm will be asked to audio record one of their treatment sessions with a study participant. These sessions must be an initial (week 1) appointment. Each audio recording will be assessed by an expert rater, who will be blinded to specific study hypotheses and allocation of the physiotherapist to the experimental or control arm. Raters will assess the overall autonomy support provided. Using a tool that will be designed specifically for this study, they will also rate the quality of the physiotherapist’s use of the 18 strategies taught during the communications skills workshops (i.e., fidelity).

### Data integrity

The research team will monitor the integrity of trial data. For physiotherapist treatment record form data, which are slightly different between experimental and control arms (i.e., 5A prompts described in the intervention section), a research investigator un-blinded to treatment allocation will perform regular data checks during data entry and provide feedback to physiotherapists regarding data omissions where necessary. For patient data, a blinded investigator will enter data within 72 hours of patient completion and follow-up with patients regarding missing data that could be salvaged. All data will be double entered, to detect and correct input errors.

### Sample size

The sample for the study was calculated using data from our recent pilot study [36]. The effect sizes observed in that investigation were *d* = .4 for adherence and satisfaction with back pain, and *d* = .5 for PA-walking behavior. These effect sizes are similar to those reported in a recent Cochrane review of trials investigating the effectiveness of interventions designed to increase adherence among chronic pain patients [16]. As a result, the estimated sample required for 80% power (α = .05) to detect a between arm effect of d = .4 in a non-clustered RCT, using a t-test, would be 156 (*n* = 78 per arm).

To account for the clustered nature of the data, we multiplied this sample size by a correction factor of 1 + (*m* − 1)ρ, where *m* was the mean expected cluster size and ρ was the anticipated intracluster correlation coefficient [38]. Based on previous research in clinical settings regarding physical activity in patients with CLBP [18,23], we estimated that the ICC would be 0.03. Assuming we recruit *m* of 22 patients per clinic, the correction factor is 1.63 for our cluster-randomized design. To account for the clustered design, the study would require 254 participants to achieve 80% power for the adherence and LBP related data.

We anticipate that approximately 15% of participants will not complete follow-up assessments [39]. Traditionally, one would account for this loss to follow up and increase the sample size by 15% to 292 participants (146 per arm). However, as our method of analysis (linear mixed models, see statistical methods section) does not require list-wise deletion, and therefore loss to follow-up is expected to have negligible impact. Indeed, Chakraborty and Gu [40] demonstrated that up to 20% of the observations can be missing without a meaningful loss of power for mixed modeling approaches. As such, our intended sample of 292 patients will provide more than 80% power to detect significant effects in these primary outcomes.

### Statistical methods

Researchers will analyze the data using a coded dataset and these individuals will not become unblinded until analysis is complete. Participants’ data will be analyzed according to their assigned arm of the study, regardless of whether they attend their physiotherapy treatment sessions or not (i.e., intention-to-treat principle).

Fidelity of the intervention will be assessed in a subsample of patients. We will employ between-arm comparisons of autonomy support ratings made by (i) patients, (ii) physiotherapists and (iii) independent, blinded raters of audio recordings. We will also compare 5A strategy use, as measured by independent, blinded raters of audio recordings.

The main study hypotheses will be explored using linear-mixed modeling with measurement occasions, patients, physiotherapists, and clinics as potential levels of the analysis. Using a dummy-coded “treatment” variable (experimental vs. control) as a predictor in the model, we will test differences both in the mean levels and the rates of change of the primary and secondary outcome variables. The primary endpoint for the analysis will be data collected at week 24. Lastly, in order to examine the process of change, we will conduct a structural equation modeling analysis, accounting for clustering effects, to test the direct and mediated relationships outlined in Figure 2.

### Adverse events

No adverse events are expected as a result of communication skills training. Patients and physiotherapists will be informed that any adverse events should be reported to and will be documented by the research team.

## Discussion points

### Potential inconveniences to the participant

As part of the study, participants will be asked to volunteer their time for the following activities that are beyond the requirements of a normal physiotherapy session; (i) baseline assessment which will require approximately 20 minutes before their initial physiotherapy appointment and five minutes after the session to complete the assessment booklet; (ii) during weeks 1, 4, 12, and 24 all participants are asked to wear a pedometer during waking hours and (iii) during week 1, five minutes will be needed to complete follow-up assessments (iv) during week 4, 12, and 24, approximately 15 minutes will be required to complete follow-up assessments.

### Limitations

Pedometers are limited as tools to measure physical activity. For example, they only provide a total number of steps in a given period, so they cannot measure the intensity of PA at a given time point or the proportion of time spent in activity above a certain intensity during a particular period. However, limited funding for this project means that it will not be possible to include more costly measures, such as accelerometers, that would likely provide more detailed data regarding physical activity.

In line with SDT tenets, the communication skills training intervention in this trial was designed to teach physiotherapists strategies that would support patients’ basic psychological needs for autonomy, competence, and relatedness. While the health behavior change model [30], upon which our study hypotheses are largely based, does not specifically include autonomy or relatedness perceptions, these constructs are theoretical mediators (along with competence) of the relationship between autonomy support and autonomous motivation [27]. As such, it would have been preferable to measure perceptions of autonomy and relatedness. Unfortunately, to the authors’ knowledge, there are no existing measures designed to tap these constructs in the physiotherapy setting or in the context of CLBP rehabilitation. We attempted to adapt measures of autonomy and relatedness from a similar context [41], but pilot data indicated significant difficulties in operationalizing these constructs. Thus, we decided not to include these measures. Further research is needed to develop scales to measures these constructs in the CLBP patient population.

## Appendix A

### General information

****·***Demographic Information:*** Each participant will have a consultation with the Research Physiotherapist to collect demographic information and medical history. (i.e. age, gender, education level, occupation and work status, past medical history, and low back pain history).

### Primary outcomes

#### Adherence

**·Sports Injury Rehabilitation Adherence Scale:** This questionnaire is designed to measure physiotherapists’ perceptions of their patient’s rehabilitation adherence. It has been shown to be a reliable scale for use in clinical physiotherapy [42].

**·Adherence to Physiotherapist’s Recommendations Scale:** To measure overall levels of adherence, we will employ the two-item adherence scale previously employed by Chan et al. [26]. The scale demonstrated acceptable internal consistency in their study involving physiotherapists.

**·Home Exercise Compliance Assessment**: To measure specific adherence to back exercise and physical activity advice we will calculate the percentage of prescribed sessions completed per week (Note: # prescribed session per week will be self-reported and confirmed from physiotherapists’ records). This measure has been previously employed in LBP studies [10].

#### Physical activity (PA)

**·International Short Form Physical Activity Questionnaire (IPAQ):** This questionnaire has produced reliable and valid scores across diverse populations [43].

#### Back pain symptoms

**·Pain Intensity Numerical Rating Scale (Pain NRS)**: The pain intensity NRS measures the participant’s average pain over the previous seven days on a 0–10 scale where 0 is “no pain” and 10 is “worst ever pain”. This scale is easy to administer and is widely used in both research and clinical practice settings where it has been shown to demonstrate good construct validity and is sensitive to change [44].

**·Pain Bothersomeness:** Following recommendations from a recent Cochrane review we will employ the “Bothersomeness Scale”, “Interference with Work Scale” and “Satisfaction with Current Symptoms Scale” from the “Core Set of Outcomes” [44].

#### Pain-related physical function

**·Roland Morris Disability Questionnaire (RMDQ):** This questionnaire consists of 24 yes/no items regarding the impact of back pain on activities of daily living. The RMDQ is used widely in low back pain studies as a standardized measure of activity limitation and has demonstrated good validity, reliability and responsiveness [45,46].

**·Patient Specific Functional Scale (PSFS):** This questionnaire is designed to assess the level of limitation on three patient-nominated activities they have difficulty performing because of their back pain. This questionnaire is anticipated to capture difficult activities that may not be represented on standardized tools. The PSFS has been shown to be a responsive measure for patients with back pain undergoing exercise-based physiotherapy treatments [47].

#### Well being

**·European Quality of Life Questionnaire (EuroQol**): The EuroQol is a standardized instrument that provides a simple descriptive profile and a single weighted health index value for health status. It is applicable to a wide range of health conditions for which it has been shown to demonstrate good validity and reliability [48].

#### Secondary outcomes

**·Health Care Climate Questionnaire**: is a six-item scale used to assess autonomy support that has demonstrated good reliability and validity [49].

**·Treatment Self-Regulation Questionnaire:** This instrument is used to assess autonomous and controlled motivation. It has demonstrated good reliability and validity across diverse health-related behaviors [50].

**·Perceived Competence Scale:** This four-item scale has consistently produced scores with good reliability and validity in relation to a variety of health-related behaviors, including PA [32].

**·Fear Avoidance Beliefs Questionnaire physical activity subscale:** This is a five-item self-report questionnaire that specifically focuses on participants’ beliefs about how physical activity affects their low back pain [51].

**·Objectively-measure physical activity:** We will measure all patients’ PA using a pedometer, which are relatively inexpensive (approximately €20) and provide basic data on daily step counts. A systematic review showed that pedometer scores correlated strongly (median r = .86) with accelerometer scores for step counts [52].

**Global Perceived Effect Scale (GPE):** The GPE is an 11-point NRS that assesses the patient’s perception of recovery. It is considered to have high face validity and is often used as the reference standard against which other subjective measures are tested when assessing their measurement properties [53].

#### Moderating variables

**·General Causality of Orientations Scale (GCOS):** This is a 17-item scale that assesses the strength of different global motivational orientations within an individual [54]. Subscales for autonomous, controlled and impersonal personality types are included.

**·Motivation to Participate Questionnaire:** to be completed by Physiotherapists in experimental arm [55], this questionnaire measures participants’ autonomous and controlled motivation for learning.

**·Expectation of Treatment Scale:** A numerical rating scale designed to assess the therapist and patient’s expectation of the intervention/treatment. It has been used widely in studies of physical interventions and shown to be a potential influencing factor in treatment outcome [56].

**·Depression Anxiety Stress Scale (DASS) Depression subscale:** The DASS includes a set of three self-report scales designed to measure symptoms of psychological distress including *depression, anxiety* and *stress,* this study will employ the seven-item depression subscale [57].

### Competing interests

DH is an Associate Editor of BMC Musculoskeletal Disorders. We acknowledge no known competing interests of any investigator on this trial.

### Authors’ contributions

CL initiated the project, contributed to the development and design of the study, developed and will implement the communication skills intervention, and will analyze data. AH contributed to the design of the study, drafted the initial version of this protocol manuscript, and will manage the project. GW contributed to the development and design of the study; he also assisted with communication skills intervention development. SM was involved in study design and the development of the CLBP management refresher workshop. NN was involved with study design and will analyze data. AM developed protocols to test intervention fidelity and will collect data. DH contributed to the development and design of the study; she also developed and will implement the CLBP management refresher workshop. All authors read and approved the final version of the manuscript.

## Pre-publication history

The pre-publication history for this paper can be accessed here:

http://www.biomedcentral.com/1471-2474/13/104/prepub
